# Automatic detection of service initiation signals used in bars

**DOI:** 10.3389/fpsyg.2013.00557

**Published:** 2013-08-30

**Authors:** Sebastian Loth, Kerstin Huth, Jan P. De Ruiter

**Affiliations:** Psycholinguistics, Faculty for Linguistics and Literary StudiesBielefeld University, Germany

**Keywords:** human robot interaction, social signal processing, intention recognition, social robotics, social signaling, action recognition

## Abstract

Recognizing the intention of others is important in all social interactions, especially in the service domain. Enabling a bartending robot to serve customers is particularly challenging as the system has to recognize the social signals produced by customers and respond appropriately. Detecting whether a customer would like to order is essential for the service encounter to succeed. This detection is particularly challenging in a noisy environment with multiple customers. Thus, a bartending robot has to be able to distinguish between customers intending to order, chatting with friends or just passing by. In order to study which signals customers use to initiate a service interaction in a bar, we recorded real-life customer-staff interactions in several German bars. These recordings were used to generate initial hypotheses about the signals customers produce when bidding for the attention of bar staff. Two experiments using snapshots and short video sequences then tested the validity of these hypothesized candidate signals. The results revealed that bar staff responded to a set of two non-verbal signals: first, customers position themselves directly at the bar counter and, secondly, they look at a member of staff. Both signals were necessary and, when occurring together, sufficient. The participants also showed a strong agreement about when these cues occurred in the videos. Finally, a signal detection analysis revealed that ignoring a potential order is deemed worse than erroneously inviting customers to order. We conclude that (a) these two easily recognizable actions are sufficient for recognizing the intention of customers to initiate a service interaction, but other actions such as gestures and speech were not necessary, and (b) the use of reaction time experiments using natural materials is feasible and provides ecologically valid results.

## Introduction

For enabling users to interact intuitively with a robotic agent, the robot system has to be able to identify and to respond to social signals appropriately. In the bar scenario, one of the most difficult challenges is to distinguish between customers who are intending to place an order and those who are not. This is complicated by the fact that bars are often dimly-lit and noisy environments with multiple customers. Detecting customers who wish to order is crucial because failing to do so is fatal for the interaction as a whole. On the other hand, inviting customers to place an order if they had no intention to do so is annoying for those customers. Thus, the system should not only detect the right signals, but also avoid false alarms. These signals could be very subtle, e.g., if a customer sits at the bar and decides to order another drink, s/he might not get up or move to another location. Using either handcrafted models for distinguishing a person who aims at initiating an interaction from people who do not wish to interact and/or deriving models from lab data, did not work as intended in the real world (Michalowski et al., 2006; Bohus and Horvitz, 2009a). The natural human-human behavior can be regarded as the gold-standard for system performance and robustness (Huang and Mutlu, 2012) and thus, the analysis of natural behavior is required for developing a model of social interaction. Our aim was to identify the signals that humans typically produce when they order in a bar from a natural data collection and to validate these signals in experiments.

## Intention recognition

Identifying whether customers would like to order requires recognizing the actions that the customers are currently performing and, secondly, understanding their communicative meaning and reason about their intention. Action recognition is the first step in the process and was defined as matching the percept of an action to a corresponding action in memory (e.g., Jeannerod, 2006), e.g., grasping a cup. Research in humans showed that mirror-neurons contribute to recognizing actions and identifying the goal of an action (Johnson-Frey et al., 2003; Iacoboni et al., 2005; Kilner et al., 2007; for review see Van Overwalle and Baetens, 2009). For example, Wurm and Schubotz (2012) compared participants observing actions in different contexts such as breaking an egg into a bowl in a kitchen and in a bathroom setting. They observed interference effects in fMRI as well as in behavioral data and argued that this reflects how suitable an action is in a given context and whether the observation fits into a plausible sequence of actions. Identifying the sequence of actions is essential for recognizing the agent's intention, but interpreting a social signal requires additional reasoning. For example, by breaking an egg the agent could indicate that s/he is taking care of preparing the meal whilst the interlocutor should complete some other task. Attracting the attention of the bartender for placing a drink order is a social intention (cf. Levinson, 1995; Van Overwalle and Baetens, 2009). For developing a set of rules that make human social intentions accessible to a robotic agent, we present a method for using natural stimuli from a real-life setting and explicitly linking action recognition and the recognition of social intentions.

The bartending robot has to rely on computer vision for recognizing the actions and posture of the customers. The research in this area focused on correctly classifying actions such as waving, walking, and running (for review see Poppe, 2010), e.g., by computing and stacking the agent's silhouettes into a space-time volume (Blank et al., 2005; Gorelick et al., 2007) or identifying relevant patches by slow feature analysis and comparing them to training data (Zhang and Tao, 2012). Also, the agent's pose (Shotton et al., 2013) and hands and faces can be identified and tracked (Baltzakis et al., 2012). That means the robotic sensors are able to extract the posture, movements and actions performed by the customers in (close to) real-time. This provides the robot system with essential information. But, as outlined above, these data have to be interpreted in a social context.

When customers try to initiate an interaction, they aim at be being recognizable to the bartender and thus, act such that their intention to place an order can be identified. Levinson (1995) referred to this kind of actions as signals. Levinson (1995) argued that the intention is the premiss of the observable actions. In terms of logic, inferring the intentions means identifying the premiss from a given conclusion (observable actions) which is logically intractable (Levinson, 1995, p. 231). This is due to the fact that there is an infinite set of premisses that would warrant the same conclusion, e.g., conclusion *p* can be drawn given *q* ∧ *p* or *q* ∧ (*q* → *p*) or *s* ∧ *p* and so on. Orkin and Roy (2007, 2009) used the behavior of several thousand players of the restaurant game for generating the actions of a virtual agent, but they showed that relying on observable behavior alone was not sufficient for deriving a meaningful structure of the interactions. However, humans can understand social signals by relying on a set of heuristics and their knowledge about the normally expected behavior (Levinson, 1995). Thus, our approach was to use the social skills of customers, bartenders and the participants in the lab experiments for deriving social capabilities for the robotic agent. We recorded real-life interactions at several bar locations. This was crucial for capturing the social behavior which would have been impossible in staged stimuli. From the recordings the customer behavior in the time span before being invited for placing orders was identified. That means the bartenders identified the customers as having the intention to place an order which enabled us to identify what the customers did when they had this intention. However, this list of behaviors could include essential behavior as well as behavior that occurred accidentally during this time. Thus, in a second step, we designed experiments for using the social intention recognition skills of the participants for identifying which actions functioned as a signal. In order to achieve this, the social scene at the bar was crucial and, thus, we selected stimuli from the natural data collection that contained the real-life social cues of the bar scene.

Transferring our results to a bartending robot required formulating a set of explicit rules. First, we have to specify which signals should trigger the robot to invite a customer for placing an order such that this robot behavior is socially appropriate. Secondly, these rules have to specify when the system should certainly not respond. This is the case if necessary signals are absent. Finally, a general preference to either invite or not to invite a customer has to be specified if the robot's sensor data are inconclusive. We review related work in the next section and introduce our natural data collection and the experiments in the following sections.

## Related work

A bartending robot is fixed at a particular position behind a bar and multiple customers can approach the system for initiating interactions (i.e., ordering drinks). In a comparable scenario, Michalowski et al. (2006) presented human-robot data collected with a robotic receptionist. Relying on proxemics (Hall, 1969), their model triggered a greeting whenever a potential interactant was sufficiently close. But people felt disturbed when they just passed by the reception desk and the robot greeted them (cf. Goffman, 1963; Michalowski et al., 2006, p. 766). This social model produced a number of false alarms due to defining the set of sufficient signals for initiating an interaction too loosely, i.e., triggering a greeting too easily. Peters (Peters, 2005; Peters et al., 2005) used eye gaze and head direction for determining the intentions of a user. This method is prone to similar errors. Thus, Sidner and her colleagues (Sidner and Lee, 2003; Sidner et al., 2005) argued that an understanding of human engagement behavior is essential. Their model relied on gaze direction, mutual face gaze, adjacency pairs and backchannels (Sidner and Lee, 2003; Sidner et al., 2005; Holroyd et al., 2009; Rich et al., 2010; Holroyd et al., 2011) and was inspired by research on human behavior in lab sessions and research on social behavior (Schegloff and Sacks, 1973). In starting an interaction, backchannels and adjacency pairs are not yet present and the model relied on eye gaze. But tracking a user's eye gaze requires a calibrated eye tracking system which is not suitable in a real-world application with naïve users. Bohus and Horvitz (2009a,b,c,d, 2010, 2011) presented a body of research relying on human-robot data collected in the wild using a static interactive platform operating as either a trivia quiz platform or a receptionist. Afterwards, the sensor data was analyzed for establishing the most predictive signals in the recordings. In these settings, the trajectory of users approaching the system was most informative in predicting the start of an interaction. The trajectory is essentially a dynamic cue and requires that the user is visible to the cameras on their way. For the bar scenario, we aimed at establishing cues that are equally applicable to customers who were already located at the bar and customers entering the scene.

Typically multiple customers are in close proximity to the bar. Thus, a method of recognizing the intention to interact which is applicable to scenarios with multiple customers is required. In contrast, most of the research on social robotics focused on single users with either one or more embodied agents (Huang et al., 2010) or at addressing the appropriate person (e.g., Jayagopi and Odobez, 2013) assuming that everybody in the scene interacts with the system. But identifying who would like to interact with the system is a major challenge. For example, Bohus and Horvitz could not cover the users' behaviors when joining the quiz game (Bohus and Horvitz, 2009a). Their model only allowed including another person in the quiz once this person was prompted by the robotic agent. In contrast, the data showed that participants joined the quiz through discussing the response options or through being prompted for advice by the active player. In other robotic agents, a number of trigger utterances were defined as a signal to initiate an interaction (Klotz et al., 2011). In contrast, we present a simple set of rules for determining the user's intention to initiate an interaction. Additionally, these rules scale to multiple users.

## Natural data collection

A video corpus of real-life customer-staff interactions at the bar was recorded in several club locations in Germany (Huth et al., in preparation). This included 105 initiations of service interactions. The time span just before the bartender invited the customers to place an order was annotated by two annotators using ELAN (Wittenburg et al., 2006). A subset of six interactions was annotated by both annotators. Both annotators identified the critical time span in all cases. The absolute differences of the start (0.33 s) and end time stamps (0.34 s) were computed and showed very good agreement compared to the average duration (35.50 s). The actions of the customers were annotated by a single annotator. The dictionaries for the customer actions were extended incrementally for covering the behavior that was recognizable to the annotator who was unaware of the current study. The summary in Table 1 counts the number of occurrences of each signal per bidding for attention. The exact timing of the actions was ignored as the analysis was limited to distinguish between highly frequent behaviors occurring in almost all interactions (e.g., *looking at bartender* in 82% or in 86 out of 105 interactions) and rare behaviors (e.g., *looking at money* in 7% or in 7 out of 105 interactions). Thus, a statistical analysis was not required.

**Table 1:** **Summary of customer behavior when bidding for attention**.

**Behavior**	**Number of interactions**	**Frequency**
**CUSTOMER BODY POSTURE AND POSITION**		
Body to bar	95	210
Head to bar	93	157
Being directly at bar	92	92
Approaching bar	44	44
Leaning on bar	12	12
Turning to bar	11	11
Further away from bar	4	4
**CUSTOMER HEAD AND LOOKING DIRECTION**		
Looking at bartender	86	246
Head gesture	11	14
Looking at money	7	13
Looking at assortment	3	3
Looking at menu	1	2
***Mimic***		
Raising eyebrows	5	9
Smiling	1	1
**CUSTOMER ATTENTION FOCUS**		
Attention to bartender	91	231
Attention to human	32	93
Attention to object	49	89
**CUSTOMER HAND MOVEMENTS**		
Holding object/bottle	17	17
Hand gesture to others	7	9
Hand gesture to bartender	4	5
**CUSTOMER SPEECH**		
Speaking to bartender	10	11
Speaking to others	21	57

The table shows the number of interactions that included a particular behavior and its absolute frequency in the attention bids.

The frequency data in Table 1 reflects the observable behavior of customers. But relying on observable behavior alone is not sufficient for extracting a meaningful structure of an interaction (cf. Orkin and Roy, 2007, 2009) nor for determining what exactly was meaningful to the bartenders (cf. Levinson, 1995). But the distinction between behavior that coincided with a response and behavior that was interpreted by the bartenders and triggered their response is crucial. For example, if customers scratched their heads frequently, this behavior would occur with a high frequency but it is not necessarily informative, i.e., head scratching and bidding for attention coincide but this does not imply a causal relationship. Thus, the natural data provides a solid base for deriving hypotheses about which signals are informative but their validity has to be demonstrated in experiments.

By definition, the potentially necessary behaviors occur in all interactions and thus, have a high frequency. All customers were directly at the bar or approached the bar. Thus, *Being directly at the bar* was identified as a candidate for a necessary signal. The remaining high frequency behaviors *attention to bartender*, *looking at bartender* and *head* and *body to bar* are similar as they indicate the person was looking at the bar. We summarize all the contributing behaviors in a single signal and refer to it as *Looking at the bar*. Robot systems are not yet able to reliably estimate the attention focus and gaze direction (without calibrating an eye tracker). However, the head and body orientation can be estimated and provide a reliable indication of where a person is looking. Thus, *Looking at the bar* (approximated by head and body direction) is another candidate for necessary signals.

The necessary signals are informative to the policy as their absence allows concluding that the customer is not bidding for attention. But for safely concluding that a customer is bidding for attention, the sufficient set of signals is required. The data in Table 1 suggests that customers successfully attracted the attention of the bartender by only *being directly at the bar* and *looking at the bar* whereas other behaviors were optional for initiating an interaction. Thus, we hypothesized that this set of two signals is sufficient.

In sum, the natural data collection suggested that the set of signals formed by *being directly at the bar* and *looking at the bar* (approximated by head and body direction) is necessary and sufficient.

## Experiment 1

The aim of this experiment was to test whether the hypothesized necessary and sufficient signals from the analysis of the video corpus were exhaustive and minimal. Additionally, we investigated how the participants recognized that a customer bid for attention. In particular, we were interested in whether participants checked the signals in a particular order and what kind of errors they committed. Both findings inform the fine tuning the robot's decision policy, e.g., if the sensor data is inconclusive the system could always invite or not invite the customer to place an order. For avoiding ambiguity, the participants of the lab experiments are referred to as *participants* and the people who participated in the natural data collection are referred to as *customers*.

The participants performed a classification task of snapshots taken from the real-life corpus. This avoided the problems associated with staging stimuli and preserved as much of the social context in the stimuli as possible. In contrast to placing a robotic system in the wild and collecting data (Bohus and Horvitz, 2009a, 2011), using real-life human-human stimuli allows investigating natural and unbiased interactions. In particular, these stimuli avoid effects of customers adapting to a specific implementation and thus, avoids potential deviations from natural behavior. A potential downside of a lab-setting is the time flow of events. When the participants in the lab are asked to respond to a snapshot, they do not experience the time constraints of a real social interaction where the response delay is typically very short. For example, research on turn-taking showed that interlocutors try to anticipate the end of a turn for a seamless conversation (De Ruiter et al., 2006). To approximate real life conditions, time pressure was introduced by limiting the response time in the experiment. The time limit was set such that the accuracy of the response did not suffer but that it effectively hindered extensive introspection.

### Methods

#### Participants

Thirty-one participants from the university population volunteered for the experiment and received €3 in exchange for their time.

#### Materials and design

For testing whether each of the two identified signals was necessary, snapshots were selected such that only one of the signals was present. Thirty-nine snapshots were selected from the natural recordings such that people stood or sat directly at the bar, but did not look at the bar or bartender (e.g., customers searching their bag or engaging in another conversation). This condition is referred to as *Being directly at bar*. Accordingly, 39 snapshots of people *Looking at the bar*, but not being directly at the bar were selected. These snapshots depicted customers who had turned toward the bar from some distance. If these signals were necessary, *no*-responses were expected in both conditions.

**Figure 1:**
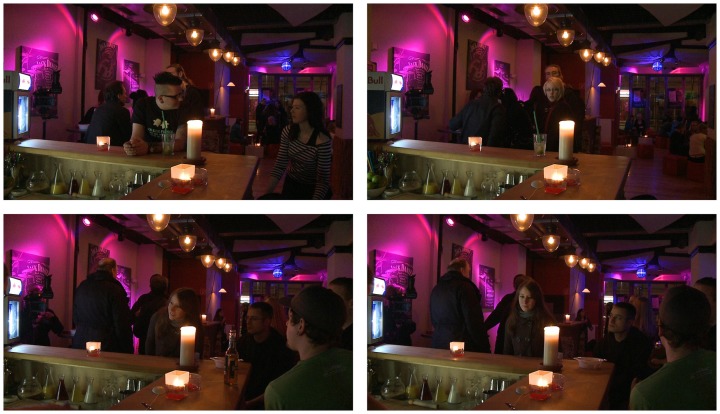
**A grid of example snapshots recorded in the “Movie,” Bielefeld. Top left**: *Being at bar* (*no*-response expected); **Top right**: *Looking at bar* (*no*-response expected); **Bottom left**: *Ordering* (*yes*-response expected); **Bottom right**: *Not ordering* (*yes*-response expected).

The experiment included two types of *yes*-trials. First, snapshots of actual orders were used and are referred to as *Ordering*. These snapshots were expected to trigger *yes*-responses irrespectively of the hypotheses. This condition formed the baseline and tested whether the participants were able to perform the task successfully. Consequently, this condition is important for assessing the validity of our experimental results. The second *yes*-condition used snapshots of customers who were not actually bidding for attention, but accidentally produced both signals. That means the snapshots showed customers producing the sufficient set of signals, i.e., they were *directly at the bar* and *looked at the bar*, but did not bid for attention. If the hypothesis was correct and these two signals formed the sufficient set, participants should be deceived into giving a *yes*-response. If some other signal was required for identifying an order, a *no*-response was expected. This condition is referred to as *Not ordering*. Only 37 of these stimuli could be identified. For balancing the number of expected *yes*- and *no*-responses, 41 snapshots of real orders were included. Furthermore, the number of expected *yes*- and *no*-responses was matched for each club location. Examples of the snapshots are presented in Figure 1.

About 11 h of recorded materials were scanned for selecting the snapshots according to the conditions of the experiment. For the baseline condition snapshots of customers placing an order were taken. The misleading condition required customers who were *directly at the bar* and *looked* there but who did not bid for attention as evidenced by the video. Finally, customers *standing/sitting directly at the bar* (but not looking there) and customers *looking at the bar* (but from a greater distance) were selected. The snapshots were double checked for ensuring that all visible customers were to be classified in the same condition, e.g., all customers in the snapshot were bidding for attention or all customers just appeared as if. This requirement ensured that the results were interpretable with regards to a specific condition.

#### Procedure

The participants were seated in front of a computer screen and provided their written consent. A gamepad was handed to the participants and its red (*no*-response) and green (*yes*-response) marked buttons were explained. The gamepads were prepared such that the participants used their dominant hand for giving a *yes*-response and the other hand for *no*-responses. All presentations on screen and the measurement of response times were controlled by DmDX (version 4.0.4.9, Forster and Forster, 2003). The task instructions were presented on screen and asked the participants to indicate by pressing the respective button whether the snapshot showed a customer who was bidding for their attention. Each trial started with a 500 ms presentation of a fixation cross which informed the participants about the upcoming snapshot. Following this, each snapshot was presented for a maximum of 3000 ms. The image disappeared as soon as the participants responded and the screen remained blank for 500 ms. If the participants failed to respond within 3000 ms, an on-screen message informed them that their response was too slow. This message was the only information about the time limit. No other feedback was provided during and after the experiment. The experimental sessions commenced with four practice trials. The snapshots resembled each of the conditions in the experiment and were not repeated in the experiment. After a self-paced break, the 156 experimental items were presented in random order. The session was interrupted by further self-paced breaks every 39 trials. The experimental session took about 15 min.

A general debriefing was provided once the experimental session was complete.

### Results

The practice items were excluded from the analysis. Out of 4836 trials, 67 (1.40%) did not receive a response (see Table 2), i.e., each participant exceeded the time limit without giving a response in about two trials on average. The number of missed responses did not differ significantly by condition [χ^2^_(3, *N* = 4836)_ = 2.307, *p* = 0.511]. All missed responses were excluded from further analyses.

**Table 2:** **Results of Experiment 1**.

**Condition**	**Expected response**	**Number of missed responses**	**Number of valid responses**	**Response score**	**Number of responses, mean response time and standard deviation**	
					***Yes*-responses**	***No*-responses**
Being directly at bar	No	14 (1.2%)	1195 (98.8%)	−0.51	292 (24%) *M* = 1558 ms	903 (76%) *M* = 1459 ms
					*SD* = 483 ms	*SD* = 493 ms
Looking at bar	No	16 (1.3%)	1193 (98.7%)	−0.47	319 (27%) *M* = 1550 ms	874 (73%) *M* = 1352 ms
					*SD* = 512 ms	*SD* = 493 ms
Ordering	Yes	16 (1.3%)	1255 (98.7%)	+0.65	1034 (82%) *M* = 1327 ms	221 (18%) *M* = 1543 ms
					*SD* = 461 ms	*SD* = 534 ms
Not ordering	Yes	21 (1.8%)	1126 (98.2%)	+0.68	947 (84%) *M* = 1313 ms	179 (16%) *M* = 1567 ms
					*SD* = 494 ms	*SD* = 524 ms

The response scores (yes-responses were scored as +1 and no-responses as −1) and times were computed for valid responses.

All responses were scored as +1 if the participant pressed the *yes*-button and −1 in case of a *no*-response independently of whether the response was correct. Thus, a perfect agreement amongst all participants that a snapshot showed a customer bidding for attention would result in a mean response score of +1.00 and that no customer bid for attention in a score of −1.00. Random responses would result in a mean response score close to 0.00. Thus, the response score provides a measure of accuracy such that values close to +1.00 or −1.00, respectively indicate a high accuracy and values close 0.00 a low accuracy. The mean values for each condition are presented in Table 2.

The response scores were analyzed using a binomial test. For each of the four conditions this showed that the response scores were significantly different from 0.0: *being directly at bar* (*Z* = 17.646, *p* < 0.001), *Looking at bar* (*Z* = 16.039, *p* < 0.001), *Ordering* (*Z* = 22.291, *p* < 0.001) and *Not ordering* (*Z* = 22.857, *p* < 0.001). In order to evaluate whether the location of the recordings and the handedness of the participants had any effect on the results a binary logistic regression was performed using condition (coding whether a *yes*- or a *no*-response was expected), handedness and a dummy recoding of the three bar locations as independent variables. The analysis showed that the condition was a statistically significant predictor of the responses (*Z* = 1367.248, *p* < 0.001). There was no statistically significant effect of handedness (*Z* = 1.882, *p* = 0.170) or the variables coding location (*Z* = 1.863, *p* = 0.172) and (*Z* = 1.724, *p* = 0.189). The difference in explained variance of the full model (Cox and Snell *R*^2^ = 0.302) and the model using condition as the only predictor variable (Cox and Snell *R*^2^ = 0.300) was negligible, thus the location and handedness were not considered in further analyses of this dataset.

The categorial responses in each condition were compared using Chi-square tests. There was no statistically significant difference between the conditions *Being directly at bar* and *Looking at bar* [χ^2^_(1, *N* = 2389)_ = 1.754, *p* = 0.185]. These two conditions received predominantly *no*-responses and the small numerical difference (see Table 2) was not statistically significant. Similarly, there was no statistically significant difference between the conditions that were predominantly associated with *yes*-responses: *Ordering* and *Not ordering* χ^2^_(1, *N* = 2381)_ = 1.245, *p* = 0.264]. A Chi-square test was also performed for comparing the level of agreement in the participants' judgment, i.e., comparing whether the proportion of expected and unexpected responses differed across conditions. Note that the expected response and the correct response were not always equal. Specifically, the majority of participants produced *yes*-responses in the *Not ordering* condition. This was compatible with our expectation, but actually a *no*-response would have been correct. In the analysis the expected *no*-responses in the *Being directly at bar* and *Looking at bar* conditions were compared to the expected *yes*-responses in the *Ordering* and *Not ordering* conditions. The test revealed a statistically significant difference [χ^2^_(1, *N* = 4769)_ = 55.100, *p* < 0.001, ϕ = 0.11]^1^ indicating a greater agreement when participants were expected to give *yes*-responses compared to the *no*-responses.

The categorial responses were also analyzed using signal detection theory. The *Being directly at bar* and *Looking at bar* trials reflected snapshots where the signal was absent and a *no*-response was expected, i.e., no customer was bidding for attention. These two conditions were combined. Similarly, the *Ordering* and *Not ordering* trials were combined (see Table 3). The results showed that *d'* was 1.62 indicating that participants performed that task well above chance. The bias was 0.31 which indicated that the participants preferred *yes*- over *no*-responses. The preference of *yes*-responses was also reflected in a greater agreement in the *Ordering* and *Not ordering* conditions than the conditions attracting *no*-responses as mentioned above.

**Table 3:** **Proportions of *yes*- and *no*-responses as a function of the presence of the two signals being at bar and Looking at bar**.

	***Yes*-response**	***No*-response**
Signals present (*yes*-response expected)	Hit	Miss
	0.832 (1981)	0.168 (400)
Signals absent (*no*-response expected)	False alarm	Correct rejection
	0.256 (611)	0.744 (1777)

The numbers in brackets show the absolute number of responses.

For analysing the response times (RTs, see Table 2), a mixed model analysis was performed using R (R development core team, 2007) and lmer in the lme4 package (Bates, 2005; Bates and Sarkar, 2007; Baayen et al., 2008). Thus, the results are reported as *F*-test. If the effect was significant at conventional levels (α = 0.05) the effect size according to Cohen (1969, p. 348)^2^ computed using G^*^Power (Faul et al., 2007) is reported. The difference in mean RT was tested using a Markov chain Monte Carlo (MCMC) simulation with 10,000 steps (Baayen et al., 2008; for examples see Brysbaert, 2007). The MCMC probability and the corresponding effect size of the equivalent *t*-test (Cohen, 1969, p. 38) are reported. The analyses included participants, items and location as a source of random variance.

The mixed model analysis tested whether the expected responses were performed faster or slower than unexpected responses. This analysis is comparable to the analysis of correct and false responses in decision experiments. There was a significant effect [*F*_(1, 4678)_ = 90.324, *f* = 0.14] indicating that expected responses were performed faster than unexpected responses (*M*_diff_ = 191 ms, *p*MCMC < 0.001, *d* = 0.38).

As with the nominal data, we were interested in whether there was a difference between the two conditions associated with the same response. The mixed model included a term for testing these contrasts within the expected and unexpected responses (the condition was a nested factor under expectation). The analysis showed a small, but significant effect of this term on RT [*F*_(6, 4673)_ = 4.506, *f* = 0.08]. The comparison of the expected *no*-responses to *Being directly at bar* and *Looking at bar* revealed a statistically significant difference (*M*_diff_ = 107 ms, *p*MCMC = 0.003, *d* = 0.22). This indicated that *no*-responses were produced faster if the customers looked at the bar from a distance compared to sitting or standing directly at the bar. There was no such difference in the unexpected *yes*-responses (*M*_diff_ = 13 ms, *p*MCMC = 0.276). Contrasting the *Ordering* and *Not ordering* conditions showed no such difference in expected *yes*-responses (*M*_diff_ = 14 ms, *p*MCMC = 0.706) and unexpected *no*-responses (*M*_diff_ = 24.0 ms, *p*MCMC = 0.901). Finally, we were interested in whether participants were faster to recognize an ordering customer compared to recognizing that nobody was about to order. For this purpose the *yes*-responses to the *Ordering* and *Not ordering* stimuli were combined and compared to the combination of the *no*-responses to the *Being directly at bar* and *Looking at bar* conditions. This analysis showed a significant difference (*M*_diff_ = 86 ms, *p*MCMC < 0.001, *d* = 0.18) indicating that spotting a customer was performed faster than establishing that no customer was about to order. The analysis of the unexpected responses across these conditions revealed no such difference (*M*_diff_ = 3 ms, *p*MCMC = 0.630).

### Discussion

The experimental design included a baseline condition using snapshots of real orders for testing the validity of the experiment. The results showed that the participants recognized that customers were bidding for attention with a high agreement (response score was 0.65, i.e., 82.5% of the responses were *yes*-responses). That means the participants were able to perform the task successfully. The signal detection analysis provided converging evidence (*d'* of 1.62). Thus, the results of this experiment are credible and interpretable. Using natural stimuli was crucial as they provided the rich social context that we investigated in this experiment. As mentioned above, recognizing the intention to order does not only require the participants to recognize an action, but importantly to interpret these actions in a specific context. This could only be achieved by using natural stimuli. But natural stimuli are less homogeneous than those generated in the lab. Specifically, each snapshot showed customers in different poses, people in the background and objects in various configurations. Understanding and interpreting the customers' intention in the natural stimuli requires more time than e.g., in controlled images with a fixed background. This resulted in relatively slow response times and large variance. However, the RTs in this experiment were comparable to other studies using natural stimuli, e.g., classification of gray-scale portrait photographs in female or male faces (O'Toole et al., 1998). In contrast, RTs in classification tasks using lab generated stimuli were much shorter (e.g., “Is this object human-made or natural?,” Gollan et al., 2005; “Is this a fruit or an animal?,” Snodgrass and McCullough, 1986). Thus, the time limit had to be set appropriately for hindering participants from extensively introspecting their intuition and allowing the participants to inspect the scene. In sum, using natural stimuli required adapting the experimental methods, but most importantly the natural stimuli reflect the real-life and increase the ecological validity of our findings. From this initial inspection of the data, we concluded that the responses were spontaneous judgments of the snapshots and that participants were able to successfully perform the task.

The analysis of the natural data collection suggested that the signals *Being directly at bar* and *Looking at bar* were both necessary for getting the attention of bar staff. If one of these signals was absent, the participants judged the snapshots as customers not bidding for attention. This provided a clear indication that both signals are necessary for bidding for attention. The same signals were also hypothesized to form the sufficient set of signals. Thus, the presence of both signals should mislead participants into assuming that the customer had the intention to order despite the fact that s/he accidentally produced this behavior. The *Not ordering* condition tested this hypothesis. The results showed that the presence of these signals was sufficiently strong to fool participants into misperceiving customers as bidding for attention who were not. Comparing the baseline and this misleading condition showed no statistically significant difference in the categorial responses and the RTs. The similarity of the results suggests that the information processed by the participants was very similar in both conditions. Thus, we concluded that *Being directly at bar* and *Looking at bar* together form the sufficient set of signals for recognizing that a customer is bidding for attention.

The analysis of the RTs suggests that participants checked these signals sequentially. The participants responded faster if the customer was located further away from the bar (*Looking at bar* condition) and they took longer if customers were directly at the bar (*Being directly at bar* condition). This suggests that participants checked whether there is somebody directly at the bar in a first step. If no customer was at the bar, one of the necessary signals was absent and this information was sufficient for concluding that a *no*-response was appropriate. But if there was a customer directly at the bar, a second analysis of the customer's body posture, head direction, engagement in other conversations and so on was required. If customers were at the bar, only this additional analysis provided the required information for evaluating whether a *no*-response was appropriate. This explains that the *Being directly at bar* condition received slower responses than the *Looking at bar* condition. The result suggests that the first process (checking the area at the bar) filtered the data for the second process (checking customers looking direction), i.e., the processes operated sequentially. But it should be noted that these results do not allow excluding that the participants assessed the presence of both signals in parallel. In this model, evaluating the head and body direction would always take more time than checking whether there are customers directly at the bar. Thus, the results of both processes would be available to the participants in sequence. The experimental data do not allow distinguishing whether there was a true sequential processing or two processes operating in parallel. However, the sequential processing has advantages for the implementation in a robotic system. The body posture is only relevant for customers who are directly at the bar. In contrast, a parallel analysis requires that the head and body direction is computed for all customers who are visible to the cameras irrespectively of their distance to the bar. Thus, the computational load is lower with sequential than with parallel processing. Consequently, the sequential account is preferable for our purposes.

The analysis of the unexpected responses showed that the participants were careful not to miss a potential order, i.e., they tried to avoid ignoring a customer. This experiment provided three sources of evidence for this conclusion. First, there was a bias of 0.31 indicating that participants had a general preference to identify snapshots as an order (giving a *yes*-response). Secondly, the participants were more accurate when a *yes*-response than when a *no*-response was expected. That means, if they made a mistake this was more likely to be a false alarm (mistaking a customer) than a miss (ignoring a customer). Thirdly, the RTs in expected *no*-responses were slower than in expected *yes*-responses. This can be attributed to an exhaustive (or at least more thorough) inspection of the snapshot when no ordering customer was identified. In turn, there was an additional effort before producing a *no*-response. These data suggest that there was a trade-off between committing false alarms (mistaking a customer) and misses (ignoring a customer). In this trade-off, the participants unconsciously avoided misses (ignoring customers) by accepting an increased rate of false alarms (mistaking customers). This could be attributed to greater social cost associated to misses than to false alarms. Thus, if the sensor data of a robotic bartender are inconclusive, the robot should invite customers to place an order. In turn, the robot's behavior would reflect that the participants in the experiment preferred false alarms (mistaking a customer) over misses (ignoring a customer).

One of the participants worked as a bartender for about 6 years, thus these data were of particular interest. Her response scores were +0.88 in the *Not ordering* condition and +1.00 with real orders. The response score for *Being directly at bar* was +0.03 and for *Looking at bar* −0.13. In sum, her results showed a strong bias to judge snapshots as ordering customers across all conditions. However, this bias resulted in perfect performance with customer having the intention to place an order. On the other hand, the performance was less ideal with customers who were not ordering. This suggests that she was careful not to miss any potential order (and sell as many drinks as possible). This reflected the data of all participants which showed that mistaking a customer is more acceptable than ignoring a potential order. In sum, the prior bar experience did not make this participant stand out in any particular way. This suggests that general social skills were involved in mastering the task rather than specific bartending skills.

In sum, two signals are necessary and together form the sufficient set of signals for identifying the intention to place an order. First, the customers position themselves directly at the bar and, secondly, look at the bar/bartender. The participants checked the presence of these signals sequentially, i.e., they applied a two-step procedure. If the participants misjudged a snapshot, the results showed that it was preferable to invite customers to order by mistake than to ignore a customer.

## Experiment 2

The timing of asking a customer for their order is important for implementing natural behavior in a robot system. An accurate account of the timing helps avoiding that customers have to wait for a system response, e.g., a time-out forces the system to wait and makes it less interactive. Consequently and in contrast to Experiment 1, this experiment used video stimuli. First, this experiment investigated whether and to what extent humans agree on when the intention to place an order is recognizable in a real-time video stream. Secondly, Experiment 1 revealed a general preference to identify customers as having the intention to interact. This experiment investigated why participants committed false alarms.

### Methods

#### Participants

Twenty-five participants were recruited in the university and received €5 in exchange for their time.

#### Materials and design

In the experiment, the participants were presented a video sequence from the same natural data collection as in Experiment 1. The participants were asked to press a button as soon as they had identified a customer's intention to place an order and to do nothing when they had not. This *go/no-go* task is similar to the bartender's task who has to respond as soon as customers bid for attention.

In total, 72 video sequences were selected. Half of the videos showed customers placing a real order. As soon as the participants responded, the video presentation terminated and the response time was recorded. Otherwise, the video presentation terminated as soon as the customer obviously interacted with the bartender and the trial was counted as a missed response (miss). The remaining 36 videos did not show customers bidding for attention. These *no-go* videos were further divided into two conditions such that half of the videos showed customers that were *Looking at the bar* from some distance and the remaining 18 videos showed customers sitting or standing *Directly at the bar*, but not looking there. The participants were not expected to respond to these trials, but to watch the videos in full length. If they pressed the response button, this was a false alarm and as with the *go* trials above, the video presentation was stopped and the time recorded.

The recording location of the videos was matched across the *go* and *no-go* trials and for the two conditions within the *no-go* trials. The duration of the *no-go* videos was matched for both conditions with an average of 18.7 s (934 frames in *Looking at bar*, 937 frames in *Being at bar*) ranging from 8.4 to 32.2 s. In the *go* trials, the video presentation stopped with the participants' response. Thus, an exact matching of the video duration could not be achieved, but the estimated response times resembled the duration and range of the video presentation in the *no-go* trials.

After the video experiment, an interview session using the videos that triggered a false alarm with the respective participant was started. In this session, the video was played back from the beginning up to the point of the original response such that the participant had the same information available as in the video experiment. The interview provided a free response text field for explaining why they thought that a customer tried to get their attention.

#### Procedure

The participants were seated in front of a computer screen and a written consent was collected. The gamepad was handed to the participants. The same buttons as in Experiment 1 were used, but all buttons were associated with the *stop*-response. The participants were free to choose which hand they would use for their response in each trial. Headphones were handed to the participants and the volume adjusted during practice. All presentations on screen and the measurement of response times were achieved through DmDX (version 4.0.4.9, Forster and Forster, 2003). The task instructions were presented on screen and asked the participants to indicate as quickly as possible if there was a customer who was bidding for their attention. They were asked to do nothing if nobody required their attention. Each trial started with the presentation of a fixation cross for 500 ms. All videos were presented in HD (1280 × 720) with the original sound. The video presentation stopped as soon as a response button was pressed and a black screen was shown for 500 ms. The experimental session commenced with four practice items. These were examples of each condition which were not used in the experiment. The short practice session was followed by a self-paced break. The experiment consisted of 72 trials with self-paced breaks every 18 trials.

For the interview part of the experiment, a self-developed JAVA-program (Java Runtime Environment, 2012) ensured that the current participant's false alarm videos were played back up to the participant's response time in the first part of the experiment (by producing the respective commands to VLC media player, 2012). The participants were asked to type in a free text field why they thought that a customer was bidding for attention. Also, they were able to replay the relevant part of the video as often as they wished. The number of interview questions was equal to the number of false alarm responses of the respective participant. The experimental session took about 20 min.

A general debriefing was provided at the end of the experimental session.

### Results

The practice items were excluded from the analyses. One response was excluded due to an extremely short RT (3 ms). Similar to Experiment 1, a response score of +1 was counted if the participant pressed the button and if they did not a −1 (see Table 4 for mean values).

**Table 4:** **Results of Experiment 2**.

**Condition**	**Response score**	**Number of**		
		***Yes*-responses**	***No*-responses**	**All responses**
Being directly at bar	−0.58	95 (21%)	387 (79%)	450
Looking at bar	−0.72	63 (14%)	355 (86%)	450
Ordering	+0.94	871 (97%)	28 (3%)	899

A binomial test on the response scores of each condition revealed that they were significantly different from 0.0: *Being directly at bar* (*Z* = 12.209, *p* < 0.001), *Looking at bar* (*Z* = 15.226, *p* < 0.001), and *Ordering* (*Z* = 28.028, *p* < 0.001). A binary logistic regression using the condition (coding which response was expected) and a dummy coding for location as independent variables showed that only condition (*Z* = 80.259, *p* < 0.001) but not location (*Z* = 3.688, *p* = 0.055) and (*Z* = 0.001, *p* = 0.993) was a statistically significant predictor of the response score. The tendency in the first variable coding location indicated that participants were more precise when stimuli were recorded in the “Ringlokschuppen” (Bielefeld) than in the “X” (Herford) or the “Movie” (Bielefeld). However, the difference in explained variance of the full model (Cox and Snell *R*^2^ = 0.063) and the model using condition as the only predictor variable (Cox and Snell *R*^2^ = 0.060) was negligible. Thus, the location of the recordings was not considered in further analyses.

A Chi-square test on the categorial responses showed that participants were more accurate in *go* than in *no-go* trials [χ^2^_(1, *N* = 1799)_ = 101.176, *p* < 0.001, ϕ = 0.24]. Analysing the categorial responses in the *Being directly at bar* and the *Looking at bar* condition revealed a statistically significant difference [χ^2^_(1, *N* = 900)_ = 7.861, *p* = 0.005, ϕ = 0.09]. This indicated that more participants erroneously pressed a *stop*-button in the *Being directly at bar* than in the *Looking at bar* condition.

The extent to which the participants agreed on when the intention to place an order was recognizable in the videos was quantified by computing the entropy of the response times (see De Ruiter et al., 2006). The RTs of correctly identified orders in the *go* condition (real orders) were assigned to 250, 500, and 1000 ms bins. For each item the maximum entropy and the entropy of all responses (Shannon, 1948, 1951) were computed according to Equation 1 associating each item with six measures of entropy (experimental and maximum data for three bin sizes, for means per condition see Table 5). By using the binary logarithm, the entropy is equal to the average number of bits required for encoding the distribution of response times according to Shannon's source coding theorem (Shannon, 1948; MacKay, 2003, p. 81). The maximum entropy reflects responses that are evenly distributed across all bins. If the experimental entropy is lower than the maximum entropy, this indicates that the responses accumulated in particular bins. Thus, the lower the experimental entropy, the greater is the agreement of the participants on when it was recognizable that a customer was bidding for attention.

**Table 5:** **Maximum entropy and entropy of correct go-responses in Experiment 2**.

**Bin size (ms)**	**Entropy of experimental data**	**Maximum entropy**
250	3.53	6.89
500	2.99	5.89
1000	2.37	4.89

$$\begin{array}{l} h=-\sum_{i=1}^{i=B}\frac{n_{i}}{N}\log_{2}\frac{n_{i}}{N}\\ h_{\max}=-\sum_{i=1}^{i=B}\frac{1}{B}\log_{2}\text{​}\left(\frac{1}{B}\right)=-\log_{2}\text{​}\left(\frac{1}{B}\right) \end{array}$$

**Equation 1**: Entropy *h* and maximum entropy *h*_max_ with *B*, number of bins; *N*, number of responses; and *n*_i_, number of responses in *i*th bin.

A pairwise *t*-test compared the experimental data and the maximum entropy for each item. The maximum and experimental entropies differed statistically significantly using the 250 ms bins [*t*_(36)_ = 17.127, *p* < 0.001, *d*_*z*_ = 2.79], 500 ms bins [*t*_(36)_ = 15.257, *p* < 0.001, *d*_*z*_ = 2.54] and 1000 ms bins [*t*_(36)_ = 13.434, *p* < 0.001, *d*_*z*_ = 2.39]. All tests indicated that the entropy in the experimental data was lower than the maximum entropy with a very large effect size. Thus, the participants showed a strong agreement in identifying when a customer bid for attention.

The interview responses were typically formed by one sentence (see Table 6 for examples). The responses were counted by the experimenter according to the signals that the participants mentioned as a trigger for their false alarm response and are summarized in Table 6. In total, 141 responses were recorded. These named a total of 174 signals, i.e., some responses named more than one signal. For example, “The customers arrived at the bar and looked at the menu” was counted in the “Being at bar” and “Reading menu” category.

**Table 6:** **Frequency of the signals mentioned in the interview responses**.

**Signal**	**Frequency**	**Example**
No order	29 (21%)	“Kein Blickkontakt, kein Bestellungswunsch” [no eye contact, no interest to order]
Eye contact/gaze to bar/bartender	38 (27%)	“Mann dreht sich nach vorne und schaut über den Tresen” [A man turns forward and looks behind the bar]
Moving to bar	21 (15%)	“Die Frau kommt zur Theke.” [The woman walks to the counter]
Changing body posture	19 (13%)	“Der Man wendet sich in der Richtung des Barmers” [The man turns around to the bartender]
Reading menu	16 (11%)	“Ich habe das sofort erkannt, weil der Gast die Karte gelessen hat. Das heisst, dass er etwas bestehlen wollte.” [I recognized this immediately, because the customer read the menu. That means that he intended to order]
Looking for/at money, holding wallet	11 (8%)	“Bringt Geld aus der Portmone raus” [Produces money out of wallet]
Being at bar	10 (7%)	“Die Gaeste sind yur Bar gekommen und haben sich die Getraenkekarte angesehen” [The customers arrived at the bar and looked at the menu]
Customers had no drink	8 (6%)	“Der Mann hat noch nichts zu trinken, gruenes shirt” [The man has nothing to drink yet, green shirt]
Pointing	4 (3%)	“Zeigebewegung der Frau” [The woman's pointing gesture]
Others	18 (13%)	“Die Frau schaut sich um.” [The woman looks about.] “Die Frau sieht suchend aus, als ob sie neu dazu gekommen ist” [The woman appears to be looking for something. As if she joined recently]

The proportion of responses mentioning this signal is shown in brackets. The German examples are presented as the participants typed them. The classification and English translation were based on the most likely interpretation.

### Discussion

The categorial response data showed that there was a great agreement amongst participants whether a customer was bidding for the attention of the bartender. This showed that participants were able to perform the task successfully. As in Experiment 1, natural stimuli were used. Especially, the video sequences including the original sound provided the social context of the bar scene. This was important because the participants had to interpret the social signals of the customers (cf. Levinson, 1995). Thus, using natural stimuli enabled us to produce results that are ecological valid and applicable in real-world settings.

The participants were less accurate in the *no-go* trials compared to the *go* trials. This finding provided converging evidence with Experiment 1 that the participants preferred committing false alarms (mistakenly assuming that a customer wants to order) over misses (ignoring a customer who wants to order). Furthermore, the accuracy was markedly lower when customers were *directly at the bar* compared to when they were further away and *looked at the bar*. This is compatible with the results of Experiment 1. Though in Experiment 1, the response times in the *directly at the bar* condition were prolonged whereas in Experiment 2 the error rate was increased. This can be attributed to an accuracy-speed trade-off. Thus, both results can be attributed to the same process of checking the sufficient set of signals. First, the participants checked whether customers were close to the bar. In the *Looking at bar* condition, this was not the case and provided sufficient evidence for not responding. But customers were close to the bar in the *Being directly at bar* condition. Thus, a second more-fine grained analysis of their body posture was required for establishing whether they were bidding for attention. This analysis introduced an additional load which resulted in prolonged response times or an increased error rate. As noted with Experiment 1, these findings could also be explained by a model relying on parallel processing. That means the processes checking each signal start at the same time. But the process checking the *Being directly at bar*-condition would terminate faster than the process analysing the looking direction of the customers. However, as noted above measuring the distance to a customer is computationally less costly than extracting their head and body posture. Thus, the sequential processing is preferable for the implementation in a robotic bartender.

The timing of the responses was of particular interest in Experiment 2. For the analysis, we computed the entropy of the RTs as suggested by De Ruiter et al. (2006). The analysis showed that the timing of the responses was predictable to the extent of 2.9 bits in the case of 500 ms bins, i.e., the number of bits required for encoding the distribution reduced from 6 bits with random data to 3 bits with the experimental data. Thus, the actual responses accumulated in particular bins indicating that the participants agreed to a large degree on when it was recognizable that a customer was bidding for attention. That means this moment was consistently identifiable in natural data.

The participants were interviewed about the cases that they erroneously identified as customers bidding for attention (false alarm) in an interview session following the experiment (see Table 6). The interview data could reveal additional cues that suggest why participants committed false alarms. It should be noted that the participants processed the stimuli in the first part of the experiment mainly automatically and thus, their responses should be treated with care. A total of 29 out of 141 responses (21%) indicated that the participant did not take the video segment as a bid for attention when attending it for a second time. That means, once the time pressure of a real-time video was removed by allowing multiple replays, the participants were more accurate in their judgment. Thus, assessing the situation in real-time made the participants more error prone. This is also reassuring that spontaneous responses were collected in Experiments 1 and 2. In the remaining interview responses, participants suggested that they identified one or more signals and that the presence of these signals made them perceive the trial as a bid for attention (false alarm). In 73 out of 141 (52%) responses, the participants identified (*looking at bar/bartender*, *being at bar*) or anticipated (*moving/turning to bar*) at least one of the signals that were tested in the experiments. That means the interview responses correcting the initial judgment and those mentioning at least one of these signals cover 102 (72%) of the responses. There was no particular pattern in the remaining responses listed in Table 6 and thus, we concluded that there was no relevant signal beyond *directly at the bar* and *looking at bar*.

## Conclusions

For enabling a bartending robot to recognize if a customer bids for attention, a natural data collection of customer and bartender behavior was recorded. These data showed what kind of behaviors customers produced. However, the observable behavior alone is not sufficient for concluding what triggered the bartender's response. Specifically, a frequently observed action could be correlated with an essential behavior. As Levinson (1995) showed, identifying which signal indicated the customers' intention to the bartenders is logically intractable. But we presented a method for exploiting the social skills of the bartenders and the participants for identifying the relevant signals. First, the time span when the participants had the intention to order was identified. This was achieved by using the bartenders' responses to customers as marker for this time span. From these data, we derived hypotheses about the relevant signals. Secondly, we tested the hypotheses in two experiments using natural stimuli. We relied on the participants' social skills to judge the situation. Thus, using natural stimuli in the experiments was essential because they provided the rich social context of a bar scene which is required for recognizing social intentions. Additionally, using natural stimuli allows eliciting responses of great ecological validity. Furthermore, the use of natural stimuli ensured the applicability of our findings. In sum, the experiments enabled us to identify which signals are necessary and sufficient for recognizing the intention to order. These findings explicate how to identify a particular intention in a rich social context and complement research on action recognition in neuroscience.

The results showed that it is necessary for customers to be directly at the bar and to look at the bar/bartender. Combined, these signals were sufficient. Furthermore, there was converging evidence that the participants checked the distance to the bar first and the looking direction in a second step. Concluding from this evidence, the robotic sensors have to accurately process customers in close proximity to the bar with regards to their body posture and head direction, but customers who are further away can be ignored. This reduces the computational demand for the vision system and in turn for reasoning about the data. If these customers look at the bar (as approximated by their body and head direction), the bartending robot should invite them for placing an order. Importantly, this method of detecting whether a customer is bidding for attention scales to multiple customers. If several customers approach the bartending robot, the two-step procedure applies to each customer. In case multiple customers wish to interact with the robotic bartender, orders have to be queued appropriately (Foster et al., 2012; Petrick and Foster, 2012).

This relatively simple policy commits to the same mistakes as humans who intuitively apply the social rules of the bar scenario. If both signals are present, this policy has to assume that a customer would like to order. The participants in Experiment 1 showed the same behavior if both signals were present in snapshots, even though the customer was not trying to get the attention of bar staff. Thus, committing these mistakes is socially appropriate rather than a fault in the policy. In sum, this policy is very robust and even the mistakes are genuinely part of the natural human behavior.

The participants showed a strong agreement on when they responded to the customers in a real-time video stream. Thus, for human participants the signals are easily recognizable from the video stream and the response occurred as soon as the signals were present. In contrast to the participants, the robotic system has to rely on sensor data. In general, the robotic sensors are capable of processing these cues in real-time (Baltzakis et al., 2012; Shotton et al., 2013), but these data can be erroneous, e.g., loosing track of a customer. However, the experimental results suggested that the robot should be tuned to minimize misses (ignoring a customer), even at the cost of an increased false alarm rate (mistaking a customer as trying to place an order). That means if the robotic bartender commits a mistake, its performance is socially more acceptable if these mistakes are false alarms rather than misses.

In summary, the results showed that two easily identifiable signals were necessary and their combined occurrence sufficient for recognizing that a customer was bidding for attention at a bar: customers were directly at the bar and looked at the bar or bartender. The participants assessed these signals sequentially starting with the customer's position at the bar and, only if applicable, the looking direction. For the implementation in a robotic agent, the sequential processing reduces the computational demand. We also showed that it is feasible to run reaction time experiments with natural stimuli, increasing the ecological validity of the findings.

### Conflict of interest statement

The authors declare that the research was conducted in the absence of any commercial or financial relationships that could be construed as a potential conflict of interest.

## Footnotes

^1^The effect size ϕ (also denoted as ϕ_*C*_ or Cramér's V) was computed according to Cramér (1946, p. 282).

^2^An effect size f of 0.1 was estimated to be a small effect, 0.25 as medium and 0.4 as a large effect.
